# Comparison of CO-RADS Scores Based on Visual and Artificial Intelligence Assessments in a Non-Endemic Area

**DOI:** 10.3390/diagnostics12030738

**Published:** 2022-03-18

**Authors:** Yoshinobu Ishiwata, Kentaro Miura, Mayuko Kishimoto, Koichiro Nomura, Shungo Sawamura, Shigeru Magami, Mizuki Ikawa, Tsuneo Yamashiro, Daisuke Utsunomiya

**Affiliations:** 1 Department of Radiology, Yokohama City University Hospital, 3-9 Fukuura, Kanazawa-ku, Yokohama 236-0004, Japan; e183077g@yokohama-cu.ac.jp (K.M.); crosspolo1027@icloud.com (K.N.); sawa0808@yokohama-cu.ac.jp (S.S.); magami.shi.ht@yokohama-cu.ac.jp (S.M.); ikawa.miz.md@yokohama-cu.ac.jp (M.I.); clatsune@yahoo.co.jp (T.Y.); d_utsuno@yokohama-cu.ac.jp (D.U.); 2 Department of Radiology, Yokohama Municipal Citizen’s Hospital, 1-1 Mitsuzawa Nishimachi, Kanagawa-ku, Yokohama 221-0855, Japan; e113044b@yokohama-cu.ac.jp

**Keywords:** artificial intelligence, deep learning, coronavirus disease 2019, coronavirus disease 2019 Reporting and Data System

## Abstract

In this study, we first developed an artificial intelligence (AI)-based algorithm for classifying chest computed tomography (CT) images using the coronavirus disease 2019 Reporting and Data System (CO-RADS). Subsequently, we evaluated its accuracy by comparing the calculated scores with those assigned by radiologists with varying levels of experience. This study included patients with suspected SARS-CoV-2 infection who underwent chest CT imaging between February and October 2020 in Japan, a non-endemic area. For each chest CT, the CO-RADS scores, determined by consensus among three experienced chest radiologists, were used as the gold standard. Images from 412 patients were used to train the model, whereas images from 83 patients were tested to obtain AI-based CO-RADS scores for each image. Six independent raters (one medical student, two residents, and three board-certified radiologists) evaluated the test images. Intraclass correlation coefficients (ICC) and weighted kappa values were calculated to determine the inter-rater agreement with the gold standard. The mean ICC and weighted kappa were 0.754 and 0.752 for the medical student and residents (taken together), 0.851 and 0.850 for the diagnostic radiologists, and 0.913 and 0.912 for AI, respectively. The CO-RADS scores calculated using our AI-based algorithm were comparable to those assigned by radiologists, indicating the accuracy and high reproducibility of our model. Our study findings would enable accurate reading, particularly in areas where radiologists are unavailable, and contribute to improvements in patient management and workflow.

## 1. Introduction

The gold standard for diagnosing coronavirus disease 2019 (COVID-19), which has caused a pandemic worldwide, is reverse transcription-polymerase chain reaction (RT-PCR) assay using nasopharyngeal mucosal swabs or oral saliva. Nonetheless, its sensitivity is inadequate at approximately 0.7, and it takes several hours to several days to obtain results [1,2,3]. On the contrary, chest computed tomography (CT) has a very high sensitivity, and diagnosis with both CT and RT-PCR has higher sensitivity [4,5]. Furthermore, while RT-PCR can detect severe acute respiratory syndrome coronavirus 2 (SARS-CoV-2), it cannot provide information regarding COVID-19 pneumonia. Representative CT images of the lungs of COVID-19 patients are characterized by multiple ground-glass opacities and crazy-paving patterns [6]. However, COVID-19 pneumonia presents various patterns on CT images depending on disease severity, making assessment difficult. Additionally, in some instances, physicians who do not specialize in managing infectious or respiratory diseases are forced to treat COVID-19 patients. Thus, these physicians, who often do not have diagnostic imaging expertise, interpret CT images to establish a diagnosis, potentially leading to misdiagnosis and inappropriate patient management.

The COVID-19 Reporting and Data System (CO-RADS), developed by the Dutch Radiological Society, grades COVID-19 pneumonia-like nature of chest CT images on a simple scale from 1 (very low) to 5 (very high) to simplify diagnostic evaluation reports [7,8,9,10,11]. This simple scoring facilitates communication between the reading physician and other healthcare providers and allows for quicker decision-making regarding treatment. However, the CO-RADS is an interpreter-dependent scoring system, and its limited reproducibility among interpreters creates problems in diagnosing, managing, and treating COVID-19 [7].

In medical imaging, artificial intelligence (AI) has progressed in recent years [12,13,14]. In particular, recent developments in AI for the analysis of COVID-19 chest CT images have facilitated distinguishing COVID-19 from other diseases exhibiting similar symptoms and recognizing signs that are sometimes missed by radiologists [15,16,17]. A previous study showed the usefulness of the CT-first triage protocol in a real-world emergency department [18]. Considering that chest radiologists may not be available for 24 h in many hospitals, CT-based triage by AI may be helpful in clinical settings. To distinguish between COVID-19, non-pneumonia, and community-acquired pneumonia, COVNet based on ResNet50 was proposed by Li et al. [15]. Their study covered 4352 chest CT scans obtained from 3322 patients. Consequently, the proposed model achieved sensitivity, specificity, and area under the curve scores of 90%, 96%, and 0.96, respectively, for the COVID-19 group. In addition, a model for detecting COVID-19 pneumonia from CT scans was proposed by Ni et al., in a study of 19,291 CT scans from 14,435 individuals [19]. The proposed model combined multiple networks for lesion detection, lesion segmentation, and lobe segmentation. Further, the model was developed to diagnose COVID-19 by analyzing the abnormal volume and the distance between the lesion and pleura. The proposed model had accuracy and sensitivity of 94% and 100%, respectively, and was superior to three radiologists. A model, fast-track COVID-19 classification network (FCONet), was proposed by Ko et al., based on VGG16 and ResNet-50 to classify COVID-19, other pneumonia, and non-pneumonia cases [20]. They included 1194 COVID-19 images, 264 low-quality COVID-19 images (for testing only), and 2239 CT scans for pneumonia, normal, and other disease cases in their study. They concluded that FCONet based on ResNet-50 outperformed other pre-trained models on an externally validated dataset of COVID-19 pneumonia images, achieving an accuracy of 96.97%. However, these methods cannot identify COVID-19 patients without COVID-19 pneumonia as positive. Therefore, to overcome this problem, Mei et al., integrated a convolutional neural network (CNN)-based COVID-19 pneumonia classification model based on chest CT findings and a multilayer perceptron-based classification model based on clinical symptoms, intensive contact history, and blood data and developed a COVID-19 pneumonia classification model that combines chest CT findings and clinical findings. They proposed a diagnostic model for COVID-19 that combines chest CT and clinical findings [16]. This model was able to correctly diagnose 17 of 25 COVID-19 patients without COVID-19 pneumonia as COVID-19-positive. An AI tool has also been proposed to automatically evaluate CO-RADS scores [21]. The system comprises the sequential application of three deep learning algorithms that perform leaf segmentation, lesion segmentation, and CO-RADS scoring, respectively. The CO-RADS score classification uses a network architecture called “Inception.” Furthermore, although this system has been compared to radiologists’ reading results, it has not been evaluated among radiologists with different years of experience. Therefore, in this study, we first developed a discriminator of CO-RADS scores for CT chest images of patients suspected of having COVID-19 using Xception, a network architecture that enables higher-level classification. Next, to validate the algorithm, we compared the AI-based CO-RADS scores with those assigned by residents and radiologists with different levels of experience.

## 2. Materials and Methods

### 2.1. Patient Population

This single-center, non-interventional, retrospective study was approved by the Institutional Review Board (approval ID: B210100046). The requirement for the acquisition of written informed consent from patients was waived owing to the retrospective nature of this study.

**Figure 1:**
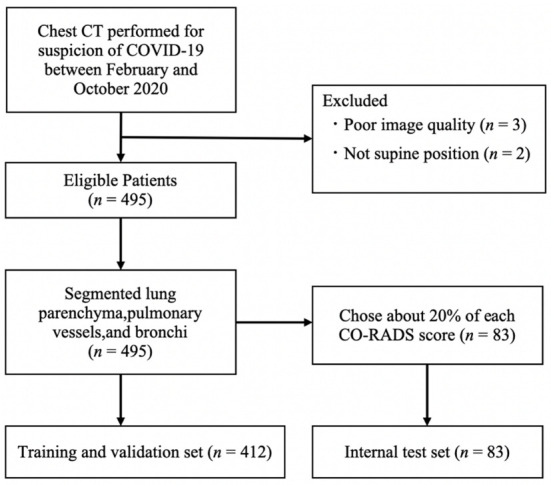
Flow diagram. Data for 500 patients who underwent chest CT for suspected COVID-19 pneumonia were collected. After exclusion, 495 eligible patients were included in the model development and evaluation. The dataset was classified into a training set (*n* = 412) and an independent patient-level test set (*n* = 83). The proportion of images with different CO-RADS scores in the test set was equivalent to that in the training set. CO-RADS; COVID-19 Reporting and Data System.

We included 500 patients who underwent chest CT imaging for clinical suspicion of COVID-19 at Yokohama City University Hospital between February and October 2020. Following the exclusion of five patients with poor-quality CT images that did not accurately classify the impaired respiratory status, the final study population comprised 495 patients. The CT images were scored by consensus among three radiologists specializing in the chest (10, 21, and 26 years of experience, respectively). The score that these radiologists assigned was used as the reference standard for the CO-RADS. A summary of CO-RADS scores is shown in Table 1. To establish and evaluate the deep-learning algorithm, 10510 CT images from 412 patients were used as training and validation data, whereas 2966 images from the remaining 83 patients were used as test data (Figure 1).

### 2.2. Data Acquisition and Image Processing

**Figure 2:**
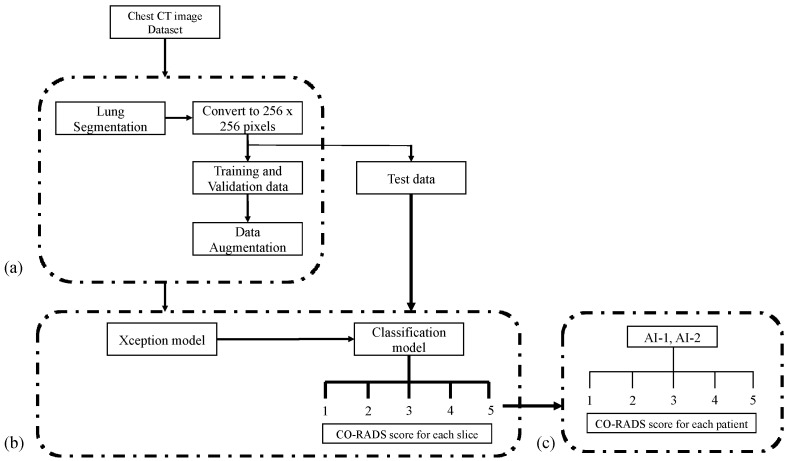
Classification workflow. (**a**) The collected chest CT DICOM images were subjected to lung segmentation using a workstation. The extracted lung fields were converted to images with 256 × 256 pixels and saved as PNG images, and the training images were augmented. (**b**) The augmented training images were subjected to the Xception model, and the test images were applied to the constructed artificial intelligence model to obtain the CO-RADS score for each slice. (**c**) The CO-RADS score for each patient was determined according to the defined method.

Chest CT scans were acquired using multidetector CT scanners with 64 (SOMATOM Definition AS+, Siemens Healthcare, Erlangen, Germany; 0.625-mm collimation, 120 kVp, and automatic tube current modulation), 80 (Aquilion Prime, Canon Medical Systems, Otawara, Japan; 0.500-mm collimation, 120 kVp, and automatic tube current modulation), or 64 (Aquilion Lightning, Canon Medical Systems, Otawara, Japan; 0.500-mm collimation, 120 kVp, and automatic tube current modulation) detector rows, with the patients in the supine position under deep inspiration. The CT image was reconstructed in the axial section with a slice thickness of 5 mm. The mean volume CT dose index was 9.2 ± 4.3 mGy, and the mean dose-length product was 362.3 ± 189.1 mGy/cm. No contrast agent was used in any of the cases. From the 5-mm slice chest CT images, the lung parenchyma, pulmonary vessels, and bronchi were automatically segmented using commercially available software (ZIO STATION 2, Ziosoft Inc., Tokyo, Japan), with manual correction. The segmented images were converted to a JPEG file with a resolution of 256 × 256 pixels (Figure 2).

### 2.3. Development of the Deep-Learning Model

**Figure 3:**
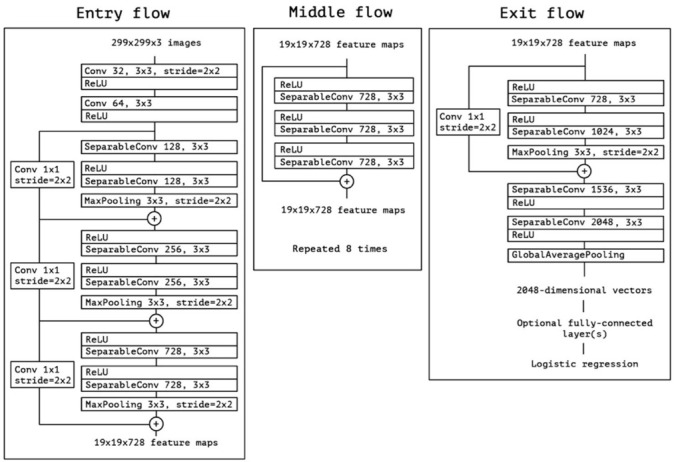
The Xception Network architecture. Reprinted with permission from ref. [22]. Copyright © 2017, IEEE.

To build a CNN-based algorithm, we used a commercially available software (Deep Analyzer, Newtech Co., Ltd., Tokyo, Japan) with the following configuration: operating system, Ubuntu 18.04.3 LTS (Canonical, London, UK); graphics processing unit, GeForce RTX 2080 Ti (NVIDIA, Santa Clara, CA, USA); and central processing unit: Core i9-9820X (Intel, Santa Clara, CA, USA). Xception was the CNN architecture used in this study. This model is a pre-trained neural network that operates on a modified depth-separable convolution, with 36 layers divided into 14 different modules. It was developed as an “extreme inception” model with a higher processing power than the conventional inception series (Figure 3) [22]. In a simple depth convolution operation, an n × n spatial convolution is performed for each channel; however, the pointwise convolution was followed by a depth convolution in this model. Xception was used to train 100 epochs, and Adam was used as the optimizer with default parameters (lr = 0.001, beta_1 = 0.9, beta_2 = 0.999, eps = 1 × 10^−7^, decay = 0, amsgrad = False [23]). All images were augmented using the following parameters: rotation range, 2.0; shear range, 0.05; and zoom range, 0.05.

### 2.4. Reading Session

The test data from 2966 images were applied to the constructed model, and AI was used to obtain the CO-RADS scores for each slice. In any individual case, the highest score among the image slices (AI-1) and the highest score in two or more consecutive slices (AI-2) were determined (Figure 2). 

The data assessed by AI were also evaluated by six independent evaluators (one medical student, two residents with three years of experience, and three senior radiologists with 8, 10, and 12 years of experience) to determine the CO-RADS score for each case.

### 2.5. Statistical Analysis

All statistical analyses in this study were performed using EZR for Windows version 1.54 (Saitama Medical Center, Jichi Medical University, Saitama, Japan) [24]. The training and testing groups were compared using the Mann–Whitney test. We calculated the intraclass correlation coefficients (ICCs) and weighted kappa coefficients for the CO-RADS scores between each observer and the reference standard and between the deep-learning algorithm and the reference standard. For every rater, the percentage of agreement between each CO-RADS score and AI-based score was calculated. Loss and accuracy were calculated when training to build the model.

## 3. Results

### 3.1. Patient Demographics

**Figure 4:**
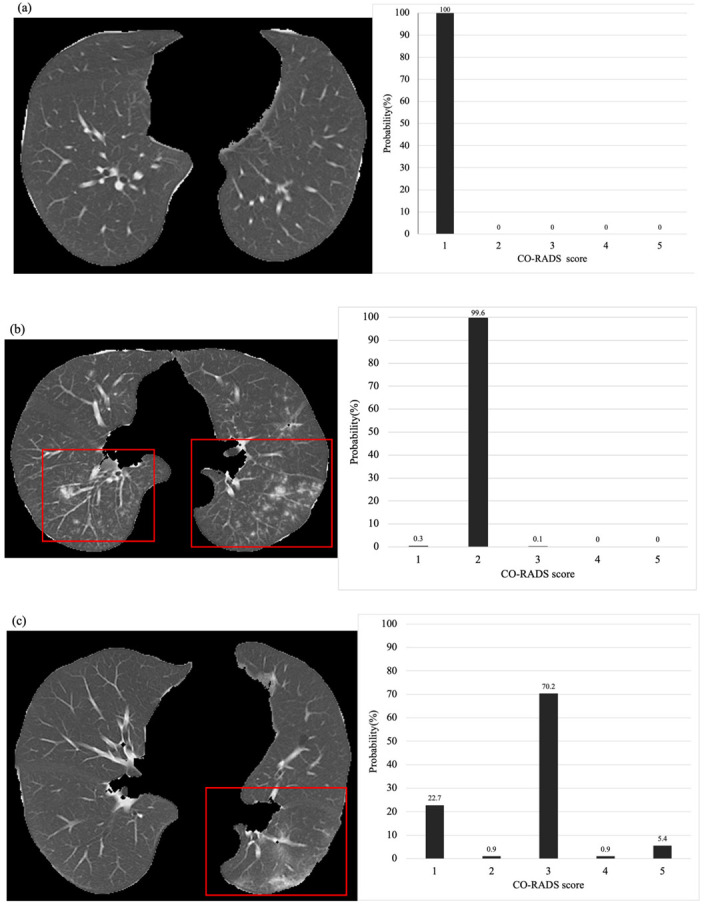

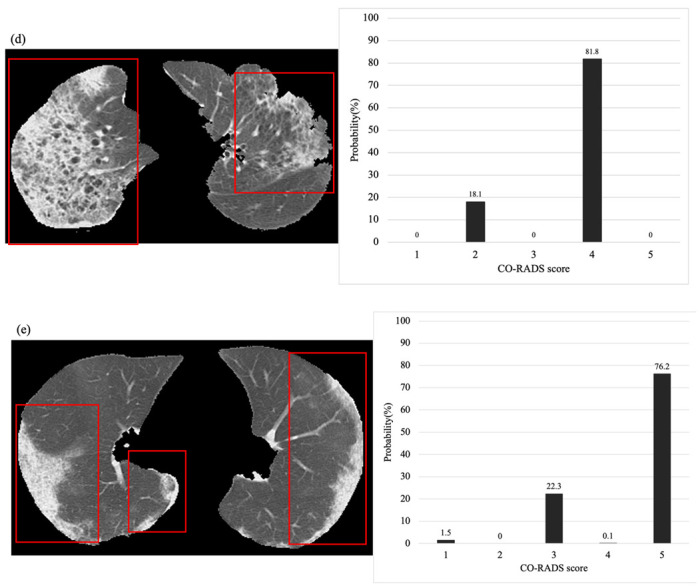
Representative output from the classification model. (**a**) The CT image shows no abnormal density in both lungs. The classification model presented a 100% probability of a CO-RADS score of 1. (**b**) CT imaging shows multiple centrilobular nodules in both lungs. The classification model presented an approximately 99% probability of obtaining a CO-RADS score of 2. (**c**) The CT images show unilateral nonspecific ground-glass opacity in the dorsal aspect of the left lung. The classification model presented an approximately 70% probability of obtaining a CO-RADS score of 3. Although a score of 3 was determined, the possibility of 1 or 5 was also suggested. (**d**) CT imaging shows bilateral subpleural predominant ground-glass opacity and consolidation and strong emphysematous changes in the background. In classification models, a CO-RADS score of 4 is most likely. (**e**) The CT image shows crazy-paving-like ground-glass opacity in the bilateral subpleural areas. The classification model also presents the highest possibility of a CO-RADS score of 5.

Table 2 summarizes the patient characteristics for the training and test datasets. Figure 4 shows the CT images of three representative cases and the probability of obtaining different CO-RADS scores based on the deep-learning classification model. There were no significant differences in the distribution of age between the training and test data (*p* = 0.06), male-to-female ratio among patients (*p* = 0.07), and CO-RADS score distribution (*p* = 0.12).

### 3.2. Deep-Learning Model and Validation

**Figure 5:**
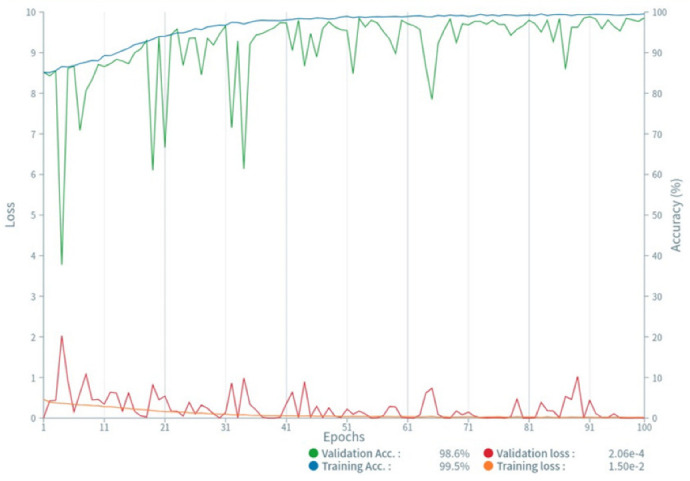
Accuracy and loss of training and validation data. The plots of training loss and validation loss decreased to a stable point, with a small gap between them.

The data of 412 patients (10,510 images) were classified into the training (90%) and validation (10%) datasets using the hold-out method, and the model was constructed and validated in 4 h. The accuracy of the constructed model was 99.5% and 98.6% for the training and validation datasets, respectively. There was no sign of overfitting, as the plots of training loss and validation loss decreased to a stable point, with a small gap between them (Figure 5). 

### 3.3. Comparison between the AI-Based and Human Evaluation of CO-RADS

The agreement between the test dataset (83 patients) and the reference standards was evaluated for the six raters and AI. AI-1 and AI-2 showed higher agreement than medical students and residents. However, AI-1 showed a slightly lower level of agreement than the certified radiologists, whereas AI-2 exhibited a higher level of agreement than the certified radiologists (Table 3). Table 4 summarizes the number of correct matches for each CO-RADS score using AI. Both AI-1 and AI-2 showed a high percentage of correct matches for CO-RADS 1, 3, and 5, but a slightly low percentage for CO-RADS 2 and 4.

## 4. Discussion

This study revealed a high agreement between the CO-RADS scores calculated using an AI-based model and those determined by experienced radiologists. The AI-derived CO-RADS scores showed a slightly higher agreement rate with the gold standard than the scores manually derived by the residents. We calculated the scores for each slice of two-dimensional data; therefore, interpretation of the score for each individual patient needs to be discussed. Calculation of the AI score from two or more consecutive slices for each patient yielded a very high concordance rate, indicating that it is a reasonable evaluation method, considering that two or more consecutive slices of pneumonia images are often evaluated in clinical practice. In contrast, when the highest score for a single slice was used, a score of 2 or higher was misinterpreted for a slice with no visually apparent abnormal concentration. This misinterpretation of a single slice by AI may be due to the learning process and should be resolved by increasing the amount of training data. The performance of AI was better than that of the radiologists when the scores of two or more consecutive slices were used as the final AI score. The discriminatory ability of CO-RADS scores 2 and 4 was low, whereas AI could accurately determine a score of 1, indicating its usefulness in diagnosing COVID-19 pneumonia. In addition, a score of 2 or more was never misjudged as 1, suggesting AI’s effectiveness in determining the presence or absence of abnormal lung shadows. However, a score of 2 was often mistaken for a score of 3, and a score of 4 was often mistaken for a score of 3 or 5. Given that the human judgment is ambiguous in cases with CO-RADS scores of 2, 3, 4, and 5, AI is not inferior to humans [25]. Nevertheless, more accurate labeling and larger training datasets are required to improve the assessment of cases with these scores.

Recently, there has been an increase in the global spread of the more infectious delta variant, leading to more severe disease [26]. Hence, the diagnosis and decision to isolate patients infected with the delta variant need to be made rapidly compared with the conventional strain, and chest CT is also important to detect pneumonia and predict severe disease [27,28]. Therefore, the model developed in this study may be significant due to its high negative predictive rate. In addition, a three-step algorithm (e.g., no pneumonia, possible pneumonia, and definite pneumonia) may be acceptable if simpler scoring is required, which is more in line with actual clinical practice.

Previous studies have reported the usefulness of AI models for diagnosing COVID-19 pneumonia and differentiating it from other types of pneumonia with high sensitivity and specificity [15,16]. Considering the inadequate sensitivity of RT-PCR tests, risk classification of abnormal chest CT shadows for COVID-19 pneumonia using the CO-RADS may be very useful in determining the isolation levels [29]. Therefore, developing an AI-based model for CO-RADS will streamline clinical practice, reduce the infection risk among healthcare workers, and ultimately improve positive diagnosis rates. The CO-RADS aids in diagnosing COVID-19 pneumonia and stratifying the risk among outpatients with a chief complaint of fever, thereby improving the workflow [7,11]. However, scoring is often dependent on the experience and ability of the reading physician. In several pandemic locations, physicians with no expertise in interpreting chest CT images have to chest CT images of patients with suspected COVID-19 pneumonia. In such situations, AI-based diagnosis can be significantly helpful. In this study, we constructed an AI model for CO-RADS scoring with a diagnostic accuracy comparable to that of radiologists.

With the widespread use of the COVID-19 vaccine, the number of infected patients is declining in some areas; nevertheless, patients with suspected COVID-19 will continue to visit hospitals on a semi-permanent basis. Even under these circumstances, chest CT plays a major role in the rapid assessment of infection risk and determination of the need for isolation and other protective measures. Furthermore, AI has high expectations to standardize risk assessment and reduce the burden on diagnostic radiologists. To improve the AI capabilities further, it is important to (a) improvise the model by adding more positive cases and those with CO-RADS scores of 2 and 4 to increase the amount of training data and (b) develop studies using data from multiple institutions.

This study has some limitations. First, the partial volume effect of 5-mm slice CT images might have made it difficult to determine the score. Second, this was a single-center retrospective study without external data validation; therefore, bias in image selection cannot be ruled out. However, we believe that the use of multiple CT machines might have mitigated the bias. Third, a high proportion of images in the dataset had a CO-RADS score of 1. This could have contributed to the higher match rate in our study, as compared to those previously reported [25]. The study population was selected during the non-pandemic period; hence, many patients visiting the outpatient clinic for fever could be non-COVID-19 patients. Despite these limitations, we believe that this study is significant because it uses a dataset closer to the real world in Japan.

## 5. Conclusions

In this study, an AI model based on the Xception Network architecture was constructed to determine CO-RADS scores for chest CT images with almost the same accuracy as radiologists. The use of this model will increase the accuracy of CO-RADS scoring for CT readings in the emergency room and enable faster triage to more appropriate treatment and care. In a follow-up study, this model could be improved with more data accumulation (e.g., multiple centers, larger sample sizes) and additional SARS-CoV-2 infection-positive cases, allowing accurate risk assessment of suspected COVID-19. In addition, we believe that the ability to omit image processing, such as the conversion of DICOM data to PNG and preprocessing, will promote the use of the model in clinical practice.

## Figures and Tables

**Table 1:** CO-RADS Scores and Summary Adapted with permission from ref. [7]. Copyright © 2020, RSNA.

CO-RADS Score	Level of Suspicion of COVID-19 Pneumonia	Summary
1	Very low	Normal or non-infectious
2	Low	Typical for other infections but not COVID-19
3	Equivocal/unsure	Features compatible with COVID-19 but also other diseases
4	High	Suspicious for COVID-19
5	Very high	Typical for COVID-19

**Table 2:** Summary of training and test datasets.

	Training Data	Test Data
Patients (*n*)	412	83
Male (*n*)	222 (54%)	48 (58%)
Images (*n*)	10,510	2966
Age		
Range (years)	4–101	6–96
Mean (years)	61	57
CO-RADS consensus score (%)		
1	249 (60%)	53 (64%)
2	56 (14%)	10 (12%)
3	72 (17%)	12 (14%)
4	25 (6%)	5 (6%)
5	10 (2%)	3 (4%)

CO-RADS; COVID-19 Reporting and Data System.

**Table 3:** Agreement with the CO-RADS score for each evaluator.

Evaluators	ICC	Mean	Kappa Value	Mean
Medical student	0.781	0.754	0.779	0.752
Resident 1	0.677		0.674	
Resident 2	0.805		0.803	
Radiologist 1	0.760	0.851	0.761	0.850
Radiologist 2	0.896		0.895	
Radiologist 3	0.897		0.895	
AI-1	0.792		0.792	
AI-2	0.913		0.912	

AI; artificial intelligence, ICC; intraclass correlation coefficient.

**Table 4:** Number of correct matches for each CO-RADS score.

CO-RADS Score	AI-1	AI-2
1	47/53 (89%)	53/53 (100%)
2	4/10 (40%)	4/10 (40%)
3	10/12 (83%)	9/12 (75%)
4	3/5 (60%)	3/5 (60%)
5	3/3 (100%)	3/3 (100%)

AI; artificial intelligence, CO-RADS: COVID-19 Reporting and Data System.

## Data Availability

Not applicable.
